# Cardioprotection of Immature Heart by Simultaneous Activation of PKA and Epac: A Role for the Mitochondrial Permeability Transition Pore

**DOI:** 10.3390/ijms23031720

**Published:** 2022-02-02

**Authors:** Martin John Lewis, Igor Khaliulin, Katie Hall, M. Saadeh Suleiman

**Affiliations:** 1 School of Physiology, Pharmacology & Neuroscience, University of Bristol, Bristol BS8 1TD, UK; 2 School of Pharmacy, Faculty of Medicine, Hebrew University of Jerusalem, Jerusalem 91120, Israel; ikbio2403@gmail.com; 3 Bristol Medical School, University of Bristol, Bristol BS8 1TH, UK; kh15742@bristol.ac.uk (K.H.); M.S.Suleiman@bristol.ac.uk (M.S.S.)

**Keywords:** ischaemia/reperfusion injury, development, mitochondria, immature heart

## Abstract

Metabolic and ionic changes during ischaemia predispose the heart to the damaging effects of reperfusion. Such changes and the resulting injury differ between immature and adult hearts. Therefore, cardioprotective strategies for adults must be tested in immature hearts. We have recently shown that the simultaneous activation of protein kinase A (PKA) and exchange protein activated by cAMP (Epac) confers marked cardioprotection in adult hearts. The aim of this study is to investigate the efficacy of this intervention in immature hearts and determine whether the mitochondrial permeability transition pore (MPTP) is involved. Isolated perfused Langendorff hearts from both adult and immature rats were exposed to global ischaemia and reperfusion injury (I/R) following control perfusion or perfusion after an equilibration period with activators of PKA and/or Epac. Functional outcome and reperfusion injury were measured and in parallel, mitochondria were isolated following 5 min of reperfusion to determine whether cardioprotective interventions involved changes in MPTP opening behaviour. Perfusion for 5 min preceding ischaemia of injury-matched adult and immature hearts with 5 µM 8-Br (8-Br-cAMP-AM), an activator of both PKA and Epac, led to significant reduction in post-reperfusion CK release and infarct size. Perfusion with this agent also led to a reduction in MPTP opening propensity in both adult and immature hearts. These data show that immature hearts are innately more resistant to I/R injury than adults, and that this is due to a reduced tendency of MPTP opening following reperfusion. Furthermore, simultaneous stimulation of PKA and Epac causes cardioprotection, which is additive to the innate resistance.

## 1. Introduction

Cardiac ischaemia/reperfusion (I/R) injury occurs during cardiac surgery [1], and is unavoidable due to the use of cardiac isolation through aortic cross-clamping and cessation of coronary blood flow. It is a major contributor to morbidity and mortality [2].

At a cellular level, the outcome from I/R injury is determined at least in part by the response of the mitochondria. Upon reperfusion of an ischaemic injury, a multimeric structure in the inner mitochondrial membrane known as the mitochondrial permeability transition pore, the MPTP, opens. The physiological function of this structure is not clear. However, it is known to have a central role in the pathogenesis of I/R injury. 

The MPTP is primed by the accumulation of Ca^2+^ and reactive oxygen species (ROS) during index ischaemia. However, it remains closed over the period of ischaemia because of acidosis. A further burst of ROS [3,4] and subsequent Ca^2+^ overload [5] trigger the opening of this pore at the commencement of reperfusion. Opening of this pore, and consequent loss of mitochondrial osmotic control, leads to swelling of the mitochondrion, disassociation of cytochrome c and is a potent trigger for apoptotic or necrotic myocyte death.

In our previous work, and that of others, we have shown that the cAMP signal transduction pathways are critical for the mediation of pharmacologically induced cardioprotective effects similar to those seen with ischaemic and temperature preconditioning [6,7,8]. For instance, the work of Lochner and colleagues [6] demonstrated that ischaemic preconditioning (IPC), a potent and well-described form of cardioprotection, causes increased cAMP activity during the preconditioning phase of IPC and that β-adrenergic signalling is a trigger for cardioprotection. Subsequently, Khaliulin et al. [7,8] demonstrated the key role of isoprenaline in pharmacologically reproducing this protection and also the central role of cAMP. 

Traditional approaches targeting the cAMP signalling pathways have encountered the problem that this approach has relied on the stimulation of β-adrenergic receptors (βAR), which may be impaired in heart failure [9,10]. Therefore, cardioprotection by activating cAMP-related signalling mechanisms bypassing the adrenoreceptors would give a significant therapeutic advantage in situations where protection is most needed. Conventionally, the biological effects of cAMP in the heart are ascribed to PKA activity [11]. However, it has become clear that cAMP also activates Epac (a guanine nucleotide exchange protein directly activated by cAMP) [12]. The cAMP/Epac pathway exists independently of and in parallel to the cAMP/PKA signalling pathway [13,14]. 

Indeed, other potent triggers of cardioprotection are known to act by signalling through PKA and Epac or downstream through PKCε, including ischaemic preconditioning. Prostaglandin E2, for instance, has been known for some time to have cardioprotective effects [15,16]. PGE2 exists in a complex regulatory web involving signalling flux through PKA and Rap1 in cardiac myocytes [17]. Indeed other, non-adrenergic modalities of cardioprotection include cAMP activity as a necessary signalling pathway, including stimuli as disparate as, for instance, dopamine and exenatide [18]. It is possible that cardioprotective signalling from many stimuli, if not all, propagates through PKA and/or Epac. 

Therefore, the use of newly available cAMP analogues that can act selectively on either PKA or Epac, or both simultaneously, represents a valuable tool to identify the involvement and the relative contribution of these cAMP sensors in cardioprotection [19] Several such analogues are available and are in common use experimentally. In this and other recent work, we have used 8-Br-cAMP-AM (8-Br, an activator of both PKA and Epac), 6-Bnz-cAMP-AM (6-Bnz, a PKA agonist) and 8-CPT-2′-O-Me-cAMP-AM (CPT, an Epac agonist). We have recently shown that simultaneous activation of PKA and Epac using these cell-permeable cAMP analogues provided strong cardioprotection against I/R injury in adult hearts [20,21]. 

However, adult hearts are known to be more vulnerable to I/R injury compared to immature hearts in animal models across a range of stages of post-natal development [22,23]. The underlying mechanisms are not fully understood, but the effects of calcium mobilisation on the MPTP have been implicated amongst the wide range of anatomical and physiological changes over the course of post-natal development [20,24,25,26]. Whether or not these protective mechanisms are effective in the immature heart is unclear. However, it is of therapeutic importance given that a large and increasing number of children each year often undergo repeated surgery for congenital heart defects. Thus, an understanding of how the young heart differs from the mature one is required in order to develop strategies for myocardial protection during surgery.

**Figure 1:**
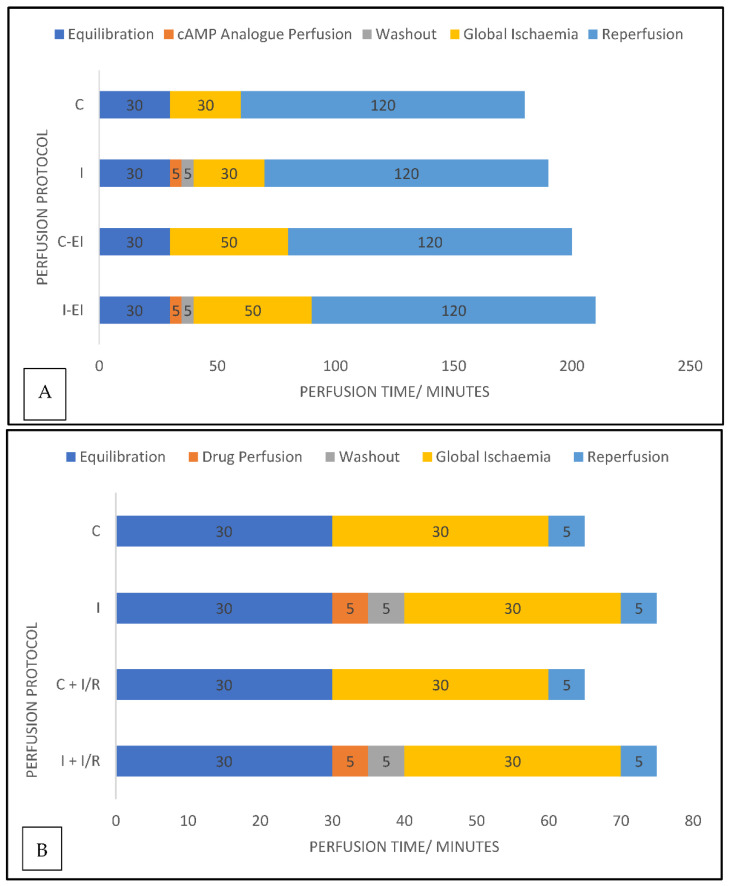
Perfusion protocols for experimentation. Graphical representations of the experimental protocols used for ex vivo heart perfusion in experiments measuring (**A**) CK activity and area of infarct size in experiments and (**B**) experimental protocols for mitochondrial isolation experiments Data labels are the durations, in minutes, of each phase of the protocol. Colour blocks indicate the phases of the experiment; blue = standard perfusion with Krebs Henseleit (KH) buffer; orange = perfusion with KH containing a drug; grey = drug washout period. C = control group; I = intervention group; EI = extended ischaemic time; I/R = ischaemia/reperfusion.

In this study, we investigated the cardioprotective efficacy of cAMP/PKA and cAMP/Epac signalling pathways in ex vivo perfused adult hearts and 14-day postnatal hearts exposed to global ischaemia/reperfusion. We used cell-permeable cAMP analogues (described in Table 1) that are selective activators of either PKA or Epac, or both, to perfuse rat hearts ex vivo. We performed measurements of biochemical indications, in the form of creatine kinase (CK) release, as well as histological indications measured as relative infarct size, to represent the scale of myocardial injury. Alterations in MPTP opening behaviour in both age groups following I/R were then investigated. The perfusion protocols for these experiments are shown in Figure 1.

## 2. Results

### 2.1. The Effects of cAMP Analogue Results in Whole Heart

#### 2.1.1. Effects of the cAMP Analogues on CK Activity in Coronary Effluent

**Figure 2:**
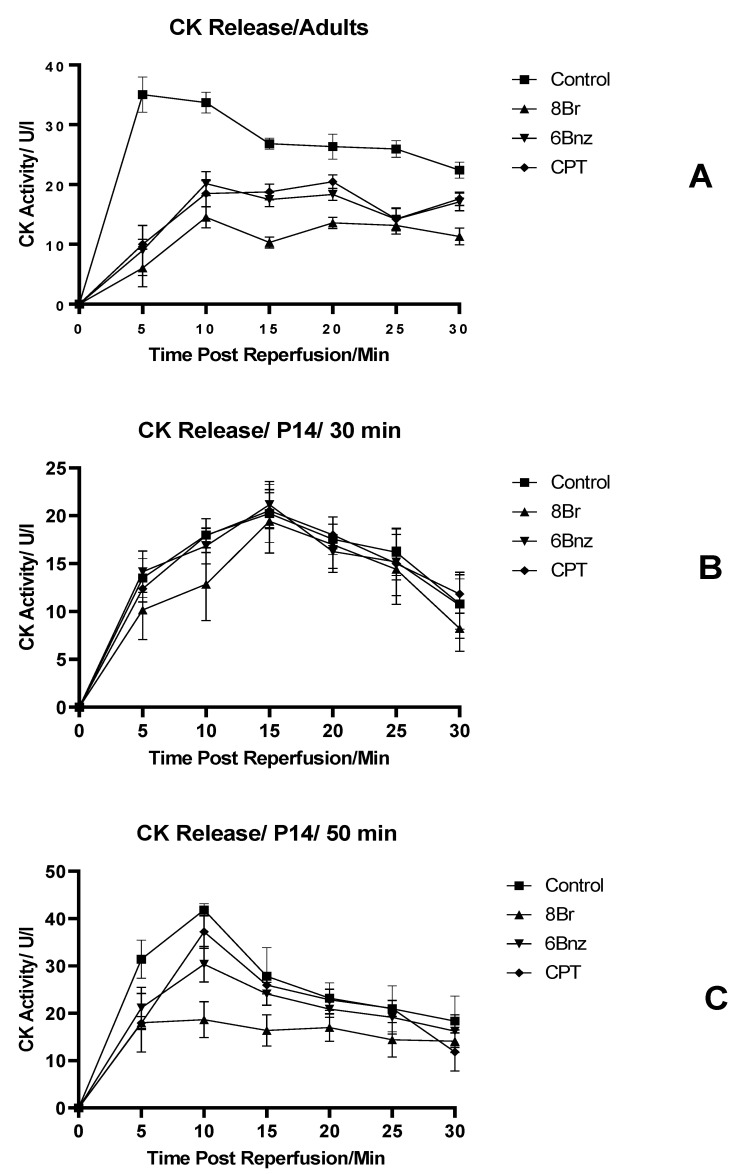
Activity of creatine kinase in coronary effluent during perfusion experiments. (**A**) Adult heart; (**B**) P14 hearts 30 min global ischaemia; (**C**) P14 hearts 50 min global ischaemia. Normalised to coronary flow rate. Error bars represent mean ± SE. *n* = 6 per group.

**Figure 3:**
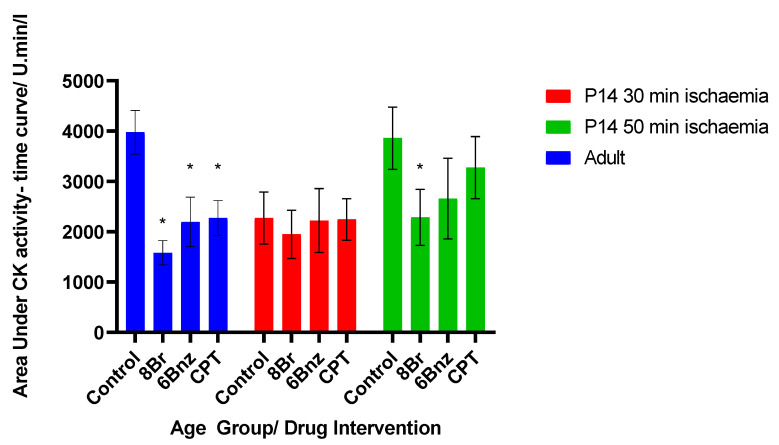
Total activity for CK in each age and treatment group for the cAMP analogue experiments. Total activity taken as estimated area under activity–time curves summed by trapezoidal addition of mean values for each time point. Bars represent mean area under curve of activity/time curves ±SE; * = *p* < 0.05 vs. same age group control. Significance tested by Student’s *t*-test. *n* = 6 per group.

Perfused adult hearts, in Figure 2A, show that CK release occurs rapidly at the beginning of reperfusion in the control group, peaking in the 5 min fraction. This is in contrast to the activity of hearts perfused with either 8-Br, 6-Bnz or CPT prior to ischaemia. All of those groups had a delayed peak, and a reduced total release vs. control. CPT and 6-Bnz had an indistinguishable effect from one another, but 8-Br produced the greatest reduction in CK release at all time points and a significant reduction in cumulative activity over the 30 min period studied (Figure 3).

Hearts from 14-day-old rats (P14) were exposed to 30 or 50 min of global ischaemia (Figure 2B and Figure 2C, respectively). The immature hearts exposed to 30 min of ischaemia did not show any difference between the control and any of the intervention groups at any of the time points studied. The peak activity level in the control was reduced to 58.6% of that seen in the adult control group, and arrived in a later fraction. Similarly, the area under the activity time curve in the P14 30 min ischaemia group was 57.1% of that in the adult group, representing a lower overall degree of injury and reduced CK activity (Figure 3).

Exposure of 14-day-old hearts to 50 min of ischaemia was performed in order to attempt to match the degree of injury between the age groups. The total area under this curve was 97.1% that of the adult hearts (Figure 3); thus, a comparable degree of injury was achieved. In this group, there was a marked reduction in CK activity in the coronary effluent in hearts exposed to any of the cAMP analogues prior to ischaemia. This was most marked with 8-Br, and also seen with 6-Bnz. CPT did not significantly reduce the CK activity.

#### 2.1.2. Effects of the cAMP Analogues on Infarct Size following Perfusion

**Figure 4:**
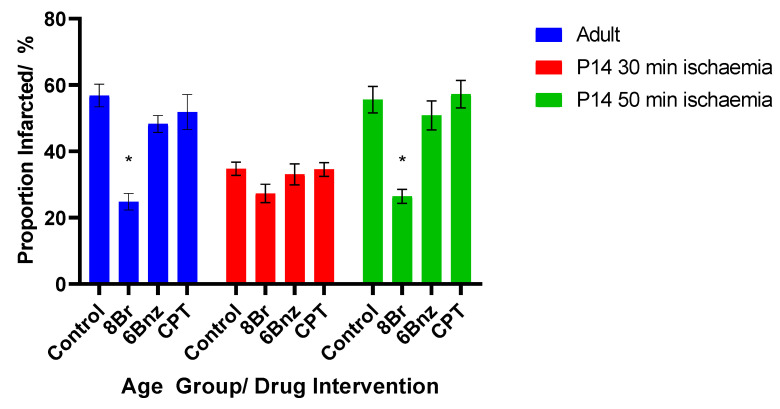
Cross-sectional proportionate infarct size vs. age group and intervention. Mean ± SE; * = *p* < 0.05 vs. same age group control. Two separate P14 groups were used with different ischaemic durations (see text). Statistical testing by Student’s *t*-test compared against same age group control. *n* = 6 per group.

A similar pattern was seen in infarct size measured following perfusion (Figure 4). In adult hearts, a 30 min ischaemic injury in the control group produced a mean infarct area of 56.8%. For the drug-treated hearts, perfusion with 8-Br, the non-selective cAMP analogue, produced a marked and significant reduction in the infarcted area to a mean of 24.8%, representing an infarct 43.7% the size of that in the control group. Neither perfusion with 6-Bnz nor CPT were associated with a significant reduction in infarct size in the adult hearts.

The group of P14 hearts subjected to an ischaemic injury of 30 min in length showed an average infarct size of 34.75%. None of the cAMP analogues in this group produced a statistically significant reduction in the area infarcted.

However, when the ischaemic time was increased to 50 min, the infarcted area increased to 55.6%, comparable to the adult hearts given a 30 min cessation of perfusion. In this group, the addition of 8-Br prior to ischaemia did produce a significant reduction in the infarct size to 26.4%. Again, perfusion with 6-Bnz or CPT did not meaningfully reduce the infarct size, so in no group perfused with either of these agents was the magnitude of the reduction in the infarct size sufficient to reach statistical significance.

### 2.2. Mitochondrial Permeability Transition Pore Inhibition

Having demonstrated the protective efficacy of simultaneous PKA and Epac activity in injury-matched P14 and adult rat hearts, we next studied whether these agents were at least in part causing cardioprotection through inhibition of MPTP opening induced by Ca^2+^ overload.

#### 2.2.1. The Effect of Isoprenaline-Induced Cardiac β-Adrenoreceptor Stimulation on MPTP Opening in Adult and Immature Hearts Not Exposed to I/R Injury

Mitochondria from similarly perfused hearts were examined in order to assess their sensitivity to swelling in response to exogenous calcium. The first group of mitochondria were perfused with control perfusate, or perfusate and isoprenaline and no ischaemia in order to demonstrate a physiological response.

**Figure 5:**
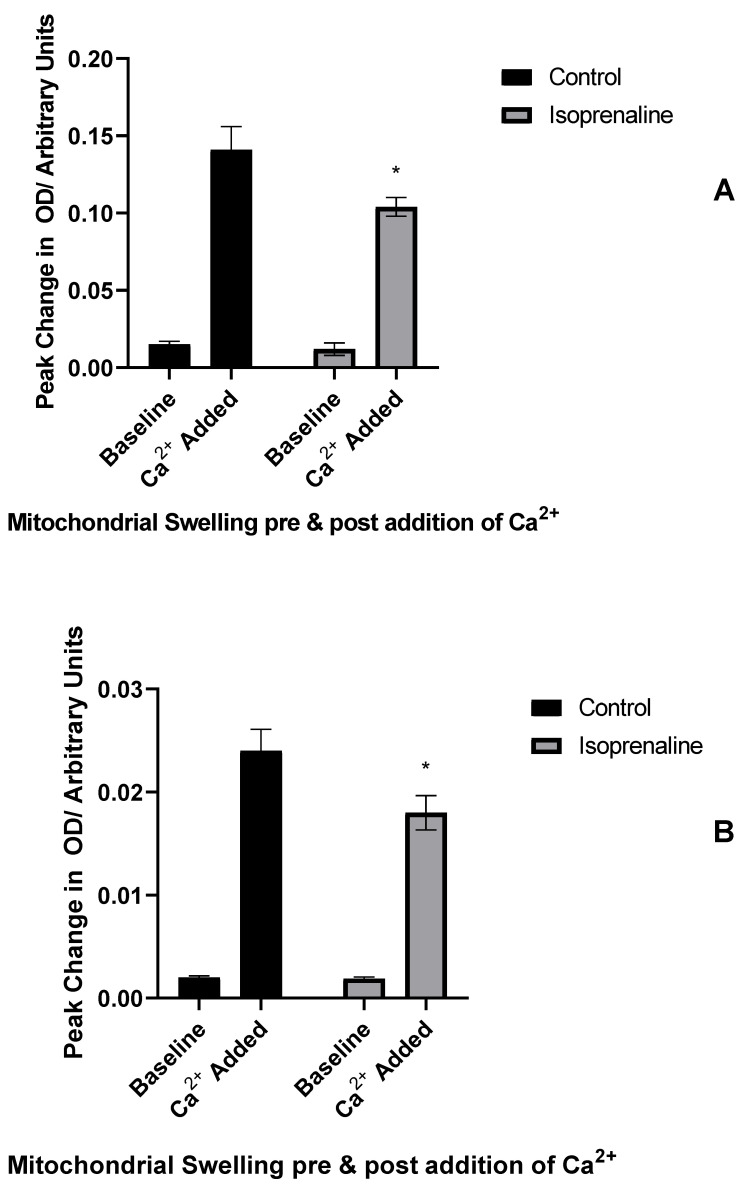
Isoprenaline-induced MPTP inhibition in hearts not exposed to I/R injury. Change in absorbance of (**A**) adult or (**B**) P14 mitochondrial extract following addition of calcium to mitochondrial suspension with or without perfusion with isoprenaline. Baseline—peak change in OD at baseline; Ca^2+^ Added—change in OD after calcium addition. * = *p* < 0.05 vs. baseline swelling in same group; data expressed as mean value ± standard error of the mean. Significance testing by ANOVA. *n* = 6 per group.

In both adult and P14 hearts, isoprenaline perfusion caused a significant reduction in mitochondrial swelling measured by a change in optical density (OD); this was true for both absolute magnitude of mitochondrial swelling (Figure 5; adults 26%, P14 33%) and maximal rate of change of absorbance (data not shown; adults 59%, P14 40%).

#### 2.2.2. Amelioration of the Effect of I/R injury on MPTP Opening by Isoprenaline or 8-Br-cAMP Perfusion

**Figure 6:**
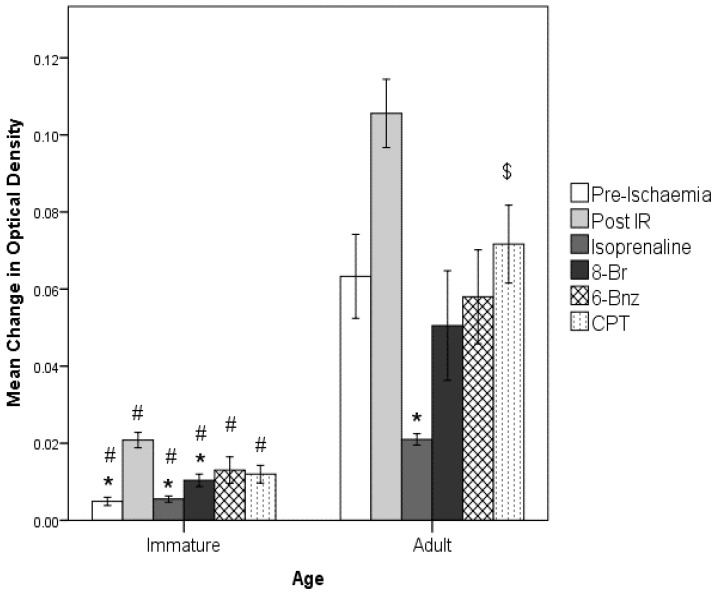
Summary of effects of interventions on MPTP activity. Error bars = 1 SEM. * = *p* < 0.05, vs. IR group in corresponding age group. # = *p* < 0.05 vs. adult age group in corresponding intervention. $ = *p* < 0.05 vs. isoprenaline in corresponding age group. Significance testing by 2-way ANOVA. *n* = 6 per age group per drug treatment.

Figure 6 shows the effect of ischaemia and reperfusion injury on MPTP opening propensity. This figure demonstrates that in both adult- and P14-derived mitochondria, a significant increase in MPTP opening was observed following I/R compared to control.

Experiments involving perfusion with the cAMP analogues, isoprenaline or none of the agents (control) were performed. Then, I/R was produced, followed by mitochondrial isolation. These results are also presented in Figure 6. Isoprenaline perfusion caused a significant reduction in MPTP opening in adult hearts to below the level seen in hearts not receiving I/R injury. This pattern, albeit at a lower magnitude, was also seen in the equivalent experiments on P14 hearts. A significant reduction in amplitude of swelling was seen following perfusion with isoprenaline, to a level comparable to the non-I/R control.

Perfusion with 8-Br, the non-selective agonist of both PKA and Epac, reduced MPTP sensitivity in adult and P14 hearts. This was indistinguishable from the uninjured control in adults. In the P14 group, there was also a significant reduction in the amplitude of swelling, although it was not reduced to the level of hearts not exposed to an I/R injury.

#### 2.2.3. Effects of CPT and 6-Bnz

The effects of the two selective cAMP analogues are also shown in Figure 6. In both the adult and P14 groups, neither agent produced a significant reduction in absolute magnitude of mitochondrial swelling. However, in the P14 hearts, both CPT and 6-Bnz produced significant reductions in the rate of swelling vs. the I/R control (6 Bnz, -−7.6 ± 1.0 × 10^−4^ OD/s *p* < 0.0005, CPT, -−8.1 ± 0.9 × 10^−4^ OD/s, *p* < 0.005), although these remained notably greater than the non-I/R control (5.1 ± 1.4 × 10^−5^ OD/s). In the adult hearts, whilst both CPT and 6-Bnz produced observable reductions in the swelling rate, only 6-Bnz reached significance; 1.2 ± 0.4 × 10^−3^ OD/s, *p* = 0.041.

## 3. Discussion

### 3.1. Combined Stimulation of PKA and Epac Provides Maximal Protection against Injury in the Adult Heart

The experiments described here show that the combined stimulation of PKA and Epac produced cardioprotection in the ex vivo perfused adult rat hearts. Consistent with previous findings [21,23], simultaneous stimulation of both parallel signalling pathways appears to be necessary for maximal cardioprotection in this model of ischaemia and reperfusion injury. Stimulation of either PKA or Epac alone does not produce the same effect as with both; an intermediate response is seen. Our previous work [7,27] demonstrated that β-adrenoreceptor stimulation was in part responsible for a cardioprotective effect following a short period of activation. However, it is known that sustained activity at this receptor family does produce deleterious consequences for the heart at both the subcellular and whole organ level, including mitochondrial dysfunction, hypertrophy, heart failure and death [28,29,30]. The potential for these untoward consequences of chronic receptor stimulation, as well as the unintended off-site consequences in the whole organism of receptor activation, mean that it is important that this work confirms prior findings of a receptor-independent pathway to cAMP linked cardioprotection.

### 3.2. The Immature Perfused Heart Shows Increased Resistance to Injury

These results in the immature heart also show the increased resistance of the developing heart to ischaemia and reperfusion injury. The developing heart does not display the same injury as that of the adult from a time-matched ischaemia/reperfusion injury; the duration of ischaemia needed to be extended from 30 to 50 min to obtain an injury, which was comparable to that shown by the adult heart after a 30 min ischaemic stimulus. This is consistent with previous work showing significant resistance to injury at this developmental stage, with an increase towards adulthood [22,23,27,31]; this observation is thought to be due to developmental changes in cardiac energetics and mitochondrial function [32] and is correlated with clinical observations of changing vulnerability to injury in the developing heart, widely recognised in the paediatric cardiac surgical community [33].

### 3.3. A Combined Action of PKA and Epac Is Needed to Protect an Immature Heart against Ischaemia/Reperfusion

Once the injury was significant enough that a hypothetical cardioprotective effect could be observed, significant reductions in infarct size and in biochemical markers of injury were seen with 8-Br perfusion. This effect was only convincingly seen with 8-Br; 6-Bnz did seem to produce a reduction in CK activity relative to control, but this effect was not matched with the change in infarct size. CPT seemed to have no significant effect.

Thus, it seems as though in the immature heart, activity of both PKA and Epac is necessary for a protective effect, just as in the adult. PKA activity alone has a small protective effect, and Epac activity alone does not seem to produce protection. Only by simultaneous activation does the maximal protective effect become apparent, implying a synergistic mechanism of action.

PKA has long been linked to cardioprotection; suggested targets through which this is mediated are, for instance, significant phosphorylation of GSK-3β [34,35], but also other molecular alterations less directly connected to the MPTP such as IKK/IκB and phosphodiesterase [36,37]. Other mechanisms previously postulated also include an interaction with mitochondrial hexokinase II, leading to MPTP inhibition [38].

The reasons and mechanisms by which Epac may have a cardioprotective effect or may potentiate the effect due to the activity of PKA are less clear. Others have not found that CPT stimulation in similar models produces an isolated protective effect [21,39]. It has been speculated that activation of PKCε, which is necessary for cardioprotection and is known to be activated in temperature preconditioning as the archetype for this phenomenon, is a downstream consequence of Epac activity [8,40].

The most obvious explanation of the marked protective effect of simultaneous activation of PKA and Epac may be that they have differing downstream effectors and so exhibit an additive effect. However, considering the extent of protection induced by the simultaneous activation of PKA and Epac compared to the much weaker effects of PKA, and especially Epac alone, it is clear that the combined effect of PKA and Epac is not simply additive. It can be suggested that the transient Epac activation magnifies the protective effect of PKA either via enhancing activation of the pathways common to both enzymes, or by activating other yet unknown signalling pathways.

One of the possible common downstream targets of PKA and Epac could be PKCε. We have previously shown that the PKA inhibitor H-89 prevented PKC activation and cardioprotection induced by temperature preconditioning, indicating that the latter activates PKC through PKA activation [8]. Moreover, our recent work has shown that PKCε (but not PKCδ) is involved in the strong cardioprotective effect of 8-Br [21]. Cazorla et al. have shown that Epac activates phospholipase C, resulting in the production of diacylglycerol and inositol triphosphate, leading to PKC activation [41]. Others have found that the β-adrenoceptor/Epac/PLC pathway specifically activates PKCε [38]. PKA, in turn, can lead to PKC activation through increased ROS production [42] and [Ca^2+^]_i_ by direct Ca^2+^-induced activation or by Ca^2+^-dependent phospholipase C [43]. Consequently, activation of PKA and Epac may converge on PKCε in the cardioprotective effect induced by cAMP. PKCε itself is known to translocate to mitochondria following other forms of protective intervention and inhibit MPTP opening [44], and so it is unsurprising that these protective interventions may do the same. The next section describes the influence of these protective interventions on MPTP opening likelihood and shows that MPTP inhibition is the likely mechanism of this cardioprotection.

### 3.4. The Immature Heart’s Mitochondria Are Less Susceptible to Ca^2+^-Induced Swelling via the MPTP Than the Adult Heart’s

These studies have shown that there is a measurable degree of swelling of the mitochondria in response to calcium in both adult and immature hearts. This degree of physiological sensitivity is demonstrably and significantly greater in the adult heart as compared to the immature heart, so although it is possible that this phenomenon may reflect unintended injury through the perfusion and isolation process, different groups exhibit this observation to differing degrees, implying that there is a physiological basis to the age-dependent sensitivity of mitochondria to Ca^2+^ overload. It is possible that developmental changes in the proteome contribute to the observed differences, both in this physiological model and in the later I/R experiments. Furthermore, the MPTP itself is even more poorly characterised in the immature heart in terms of its components and structure than in the adult; it may be that differences in these characteristics could account for the observed differences.

The overall physiological role of the MPTP in contexts aside from ischaemia and reperfusion injury remains unclear, and further studies on the MPTP in its intracellular environment are needed to address this.

### 3.5. Ischaemia and Reperfusion Injury Sensitises the MPTP to Ca^2+^ in Both Immature and Adult Hearts

Both age groups demonstrated a significantly larger swelling response to calcium following exposure to I/R injury. The MPTP is described as being sensitised to opening stimuli during reperfusion, which is thought to be a consequence of the influence of reactive oxygen species. Therefore, these observations are expected in the context of prior work [45], but do confirm this work as a valid model of the known effects of reperfusion injury.

### 3.6. The MPTP Is less Likely to Open in the Immature Heart Exposed to Injury Than the Adult Heart

Although the proportionate change in sensitivity to MPTP opening after I/R is greater in the immature control hearts, the absolute magnitude of this swelling response is less than a fifth that seen in the adult heart. A similar pattern is seen concerning mean rates of swelling. This is strikingly similar to the vulnerability pattern to whole-heart global ischaemia and reperfusion injury, described as bell-shaped or biphasic wherein vulnerability is thought to decrease from birth to 2 weeks post-natal age in rats before rising again to a peak at adulthood [23,46]. It is therefore appealing to suggest that at least one of the factors underpinning this change in vulnerability is the changing behaviour of the MPTP with age [26]. Why the MPTP should change with age is not clear; the structure of this pore is still a matter of debate, and its constituents are still not comprehensively clear [46,47]. The structural knowledge that does exist is from work in adult tissues, and so cannot be assumed to remain consistent in the immature heart. It is further possible that mitochondrial morphology differs in the immature heart from that of the adult, which may have further consequences for the vulnerability to swelling.

### 3.7. MPTP Inhibition Is Replicated by cAMP Analogues but Requires PKA and Epac Synergy

Prior perfusion of hearts with 8-Br reduces the sensitivity to swelling of both adult and immature mitochondria to almost, but not quite, the level seen with isoprenaline. This study was not powered or intended to be seen as a non-inferiority study between these two compounds. However, it may be that isoprenaline acting at the receptor level stimulates other occult pathways of signalling in parallel, or that there is a different amplitude of response along the downstream communication channels.

This study has, however, demonstrated that at a mitochondrial level, a cAMP analogue stimulating both PKA and Epac produces protection against ischaemia and reperfusion injury. This effect is not seen in either age group following stimulation with either CPT or 6-Bnz alone, where the response is not significantly different from that following I/R alone. This may imply that the reduction in sensitivity is due to a synergistic effect of the action of PKA and Epac. It is, however, also possible that the protection depends on a mechanism not stimulated by either of these agents but by an off-target effect of 8-Br.

## 4. Materials and Methods

### 4.1. Animals Used for Experimentation

All of the experiments used male Wistar rats obtained by the University of Bristol Animal Services Unit on our behalf. A total of 96 animals were used in the experiments described here. Adult rats were obtained from the Charles River Laboratory (Wilmington, MA, USA), and the immature rats of the same species were bred in-house by the University of Bristol Animal Services Unit.

The adult rats were allowed one week acclimatisation in the animal housing following arrival before use in these studies, and the immature rats were maintained in the same facility since birth. The housing was purpose-built and temperature-controlled; the animals were given free access to standard food and water and no discrimination between experimental groups was made for housing. Six animals were housed at a time and were randomised to groups within that block. Both adult male and immature (14 days post-natal) rats were used. The adult rats had a mean weight of 337.9 g (SD 48.9), and the immature rats had a mean weight of 31.4 g (SD 3.7).

The group size for each control and intervention group in both the CK release/infarct size experiments and mitochondrial swelling experiments was 6 per group. Therefore, 48 perfusion experiments were completed for the CK release and infarct size study. For the mitochondrial experiments, data from 72 animals were included. A single heart was perfused at a time; experiments were performed on a temperature-controlled apparatus at the same time of day each day in order to minimise environmental influence.

### 4.2. Extraction of Hearts

In all cases, whole hearts were extracted from adult (c. 300 g) and immature (14-day-old) male Wistar rats. Animals were killed by cerebral concussion followed by cervical dislocation. A sternotomy incision was then made followed by reflection of the ribs, and the heart was lifted out of the thorax. The aorta was cut along the descending portion, and the heart was immediately placed in Krebs–Henseleit buffer chilled to 4 °C.

### 4.3. Langendorff Perfusion

The hearts were lifted onto a perfusion cannula (16G adult/24G immature) by the aorta, and fixed in place through a suture over the overlap of aorta and cannula. They were contained within an insulated glass jacket and maintained at normothermia.

A constant-flow perfusion methodology was used. Krebs–Henseleit (KH) buffer at 37 °C was pumped through the apparatus at a rate of 10 mL/h for adult hearts, and 4 mL/h for immature hearts. The buffer was oxygenated with 95% O_2_/5% CO_2_. Additional reservoirs of KH buffer with the addition of the drug(s) under investigation were also included in the circuit; perfusion was switched to these reservoirs for the duration indicated in the experimental protocol.

### 4.4. Experimental Protocols

The experimental protocols for perfusion of hearts used for cAMP analogue perfusion and corresponding controls with CK activity and histological analysis are shown in Figure 1A. The control hearts in this group also had a 30 min equilibration period, followed by a 30 min period of global ischaemia, and subsequently, a 120 min reperfusion period; intervention hearts were also given a 5 min period of perfusion with a cAMP analogue followed by a 5 min washout period. The agents chosen were selective agonists of either Epac or PKA, or non-selective between these intermediaries (known as 8-Br/6-Bnz/CPT, described fully in Table 1). These agents were supplied by Biolog Life Science Institute (Bremen, Germany). We used 10 µM CPT, with 5 µM 8-Br and 6-Bnz based on prior work in our group demonstrating equipotency.

Perfusion was followed by a 5 min washout period and then a 30 min period of ischaemia with a 120 min period of reperfusion (*n* = 6 per age group per drug treatment + control). An additional group was added post hoc of immature (P14; *n* = 6 per drug treatment + control) hearts exposed to a 50 min duration of global ischaemia in order to match the degree of injury shown in adult hearts.

### 4.5. Measurement of Creatine Kinase Activity

Coronary effluent was collected from all the perfused hearts and assayed for creatine kinase (CK) activity. Samples were collected prior to perfusion and at 5-min intervals following perfusion from hearts used for the assessment of infarct size in order to obtain samples during the period of peak injury. Determination of CK activity was performed using a kit available from Randox (Crumlin, Northern Ireland). This assay uses the rate of formation of NADPH from NADP, dependent on ATP production catalysed by CK, as a proxy for CK activity. The rate of absorbance change in the reaction mixture at 340 nm was measured and then converted into an estimate of CK activity.

### 4.6. Measurement of Infarct Size

Following perfusion, each heart was sliced and stained with 1% triphenyl tetrazolium chloride (TTC) for 20 min at 37 °C, then fixed with 4% formaldehyde at 4 °C overnight; this method resulted in infarcted areas appearing pale and viable tissue stained a deep red colour. Each heart was sliced into 5 transverse sections, and each slice was scanned on both sides to allow digital estimation of the area infarcted, expressed as a proportion of the size of that slice. Estimation of the infarcted area was digitally performed using ImageJ (National Institutes of Health, Bethesda, MD, USA). The mean value across these sections was then taken as the area infarcted for that sample.

### 4.7. Perfusion Protocols for Mitochondrial Isolation Experiments

Two sets of experiments were performed, shown in Figure 1B. The first investigated the response of mitochondria isolated from hearts not exposed to I/R injury to isoprenaline, a non-selective β adrenoreceptor agonist (adult control *n* = 6, intervention *n* = 6; P14 control *n* = 6, intervention *n* = 6), and a second set of experiments examined the response following isoprenaline or PKA/Epac agonist and a 30 min global ischaemic injury followed by a 5 min reperfusion period. The cAMP analogues used in these experiments were the same as for the experiments for CK activity and infarct size; they are shown in Table 1.

### 4.8. Mitochondrial Isolation

At the end of the perfusion protocols, the hearts were removed from the aortic cannula and placed into chilled KH buffer. They were then homogenised using a Polytron Kinematica probe, and the resulting suspension was centrifuged at 2000× *g* for 90 s in order to separate the cell debris.

The supernatant from that step was then removed and centrifuged again at 10,000× *g* for two iterations of 5 min, with the supernatant removed and pellet resuspended each time. The resulting suspension was considered to be a preparation of isolated mitochondria.

These mitochondria would be of variable concentrations due to the differing masses of the hearts used initially, and the varying proportion of mitochondria within those hearts. The concentration was then estimated through a Bradford essay, and this information was used to normalise at 0.2 mg/mL in the mitochondrial swelling buffer.

### 4.9. Mitochondrial Swelling Assay

The swelling of these mitochondria in response to 1 mM Ca^2+^ was then assessed in a spectrophotometer (Evolution 201, Thermo Scientific, Waltham, MA, USA) at 520 nm. A baseline recording at 37 °C was made before the addition of Ca^2+^ to the solution. The recording was then restarted and continued for at least 300 s or until any change in absorbance had ceased in order to measure the change in absorbance characteristics that accompanies swelling of the mitochondria as a consequence of MPTP opening triggered by the addition of Ca^2+^. The maximal change in absorbance amplitude was then calculated as a measure of effect size.

### 4.10. Statistical Analysis

Statistical testing for all of the results presented in this work was performed using SPSS version 25.0 (IBM Corp., Armonk, NY, USA). All data sets for CK release, infarct size and mitochondrial swelling were tested for statistical normality by means of the Shapiro–Wilk test. For the CK release and infarct size data, Student’s *t*-test was used to estimate significance, with the null hypothesis rejected at *p* < 0.05. For the mitochondrial swelling data, two-way ANOVA with Tukey’s post hoc test of significance was used to assess the differences between the age groups and drug treatments studied. Again, the null hypothesis was rejected at *p* < 0.05.

## 5. Conclusions

This work shows that the immature heart is innately more resistant to I/R injury than the adult in ex vivo perfusion models, not only at the whole organ level, but also at the level of isolated organelles, inferring a role for the MPTP in the mechanism for that resistance. Adult and immature hearts show an inducible, similar cardioprotection after the activation of both PKA and Epac; this persists despite the increased innate resistance of immature hearts to I/R injury. These data demonstrate the central role of the MPTP in cardioprotection, and encourage further detailed study of its behaviour and broader mitochondrial function in the immature heart in order to elucidate the mechanism of innate and inducible protection.

## Figures and Tables

**Table 1:** List of cAMP analogues used in these studies.

cAMP Analogue	6-Bnz-cAMP-AM (6-Bnz)	8-CPT-2′-O-Me-cAMP-AM (CPT)	8-Br-cAMP-AM (8-Br)
Function	PKA activator	Epac Activator	Activator of both
Perfusion Concentration	5 µM	10 µM	5 µM

## Data Availability

Data are available on request from the corresponding author.
